# A global indicator of species recovery

**DOI:** 10.1111/cobi.70077

**Published:** 2025-06-09

**Authors:** H. Resit Akçakaya, Michael Hoffmann, E. J. Milner‐Gulland, Molly K. Grace, Barney Long

**Affiliations:** ^1^ Department of Ecology and Evolution Stony Brook University Stony Brook New York USA; ^2^ IUCN Species Survival Commission Gland Switzerland; ^3^ Conservation Programmes Zoological Society of London London UK; ^4^ Department of Biology University of Oxford Oxford UK; ^5^ Re:wild Austin Texas USA

**Keywords:** conservation impact, Convention on Biological Diversity, Global Biodiversity Framework, Green Status of Species, Red List Index, species recovery, Convenio sobre la Diversidad Biológica, estado verde de las especies, impacto de la conservación, índice de la lista roja, Marco Mundial de Biodiversidad, recuperación de especies, 《生物多样性公约》, 《全球生物多样性框架》, 物种恢复, 物种绿色状况, 红色名录指数, 保护影响

## Abstract

Monitoring progress toward meeting global biodiversity goals involves several indicators, including, at the species level, the International Union for Conservation of Nature (IUCN) Red List Index (RLI) and the Living Planet Index (LPI). However, at present, there is no indicator specifically for tracking species recovery, despite this being enshrined in the mission of the Convention on Biological Diversity's Kunming–Montreal Global Biodiversity Framework (GBF). The IUCN recently adopted the Green Status of Species (GSS), a global standard for measuring species recovery and for assessing the role played by conservation in species recovery. An index based on GSS has been adopted as an indicator for multiple elements of GBF. However, a methodology underpinning the index itself has not previously been published or elaborated. We have therefore developed the Green Status Index of Species Recovery (GSI) for use as a global indicator of progress toward species recovery. We devised GSI to reflect the uncertainties of the underlying GSS assessments and developed methods to disaggregate its global value to reflect the contribution of each country to the recovery of the species within its borders. Overall, we designed the GSI to exhibit key attributes of an effective global indicator, including an explicit objective aligned with global biodiversity goals and a sound methodological basis. The GSI complements existing indicators, such as RLI and LPI, because it fills an important niche in measuring biodiversity trends, going beyond extinction risk and population abundance. As a test, we applied the GSI to a set of species and found that these species were less than halfway to full recovery and moved farther away from full recovery since the mid‐20th century. Although the deployment of GSI for complete taxonomic groups will require a considerable scaling up of effort, a sampled approach is feasible and can be operational by 2030.

## Abstract (es)

Un indicador global de la recuperación de especies

**Resumen:**

El seguimiento de los avances para lograr los objetivos mundiales de biodiversidad incluye varios indicadores, entre ellos a nivel de especies: el Índice de la Lista Roja (IRL) de la Unión Internacional para la Conservación de la Naturaleza (UICN) y el Índice Planeta Vivo (IPV). Sin embargo, en la actualidad no existe ningún indicador específico para el seguimiento de la recuperación de especies, a pesar de estar consagrado en la misión del Marco Mundial de Biodiversidad (MMB) de Kunming‐Montreal del Convenio sobre la Diversidad Biológica. La UICN adoptó recientemente el Estado Verde de las Especies (EVE), una norma mundial para medir la recuperación de las especies y evaluar el papel de la conservación en su recuperación. Se ha adoptado un índice basado en el EVE como indicador de múltiples elementos del MMB. Sin embargo, no se ha publicado ni elaborado previamente una metodología que sustente el índice. Por ello desarrollamos el Índice de Estado Verde de Recuperación de Especies (IEVE) para utilizarlo como indicador global de los avances hacia la recuperación de las especies. Diseñamos el IEVE para que refleje las incertidumbres de las evaluaciones subyacentes del EVE y desarrollamos métodos para desglosar su valor global de modo que refleje la contribución de cada país a la recuperación de las especies dentro de sus fronteras. En general, diseñamos el IEVE para que muestre los atributos clave de un indicador global eficaz, incluyendo un objetivo explícito alineado con los objetivos globales de biodiversidad y una base metodológica sólida. El IEVE complementa los indicadores existentes, como el IRL y el IPV, porque cubre un nicho importante en la medición de las tendencias de la biodiversidad, yendo más allá del riesgo de extinción y la abundancia de las poblaciones. Como prueba, aplicamos el IEVE a un conjunto de especies y descubrimos que estas especies se encontraban a menos de la mitad del camino hacia la recuperación total y desde mediados del siglo XX. Aunque el despliegue del IEVE para grupos taxonómicos completos requerirá un aumento considerable del esfuerzo, un enfoque basado en muestreos es factible y puede ser operativo para 2030.

## 物种恢复的全球指标

【摘要】

监测实现全球生物多样性目标的进展涉及多个指标, 包括物种水平的国际自然保护联盟(IUCN)红色名录指数(Red List Index, RLI)和地球生命力指数(Living Planet Index, LPI)。然而, 尽管物种恢复被正式列入了《生物多样性公约》《昆明‐蒙特利尔全球生物多样性框架》的使命之中, 但目前还没有专门用于追踪物种恢复的指标。IUCN最近通过了“物种绿色状况(Green Status of Species, GSS)”, 作为衡量物种恢复和评估保护对物种恢复的作用的全球标准。一个基于GSS的指数已被采纳为评估《全球生物多样性框架》多个要素的指标。然而, 支撑该指数的方法尚未发表或阐述。因此, 本研究开发了物种恢复绿色状况指数(Green Status Index of Species Recovery, GSI), 来作为物种恢复进展的全球指标。我们设计了GSI来反映GSS评估的不确定性, 并制定了分解其全球水平数值的方法, 以反映每个国家对其境内物种恢复的贡献。总之, 我们设计的GSI展现了一个有效全球指标的关键属性, 包括与全球生物多样性目标一致的明确目的和健全的方法论基础。这一GSI指数是对现有指标(如RLI和LPI)的补充, 因为它在衡量生物多样性趋势方面填补了一个重要的生态位, 超越了灭绝风险和种群丰度的范畴。我们还将GSI应用于一组物种作为测试, 并发现这些物种自20世纪中期以来, 还没有达到完全恢复的一半, 且距离完全恢复越来越远。尽管在所有类群中采用GSI指数仍需更大程度的努力, 但部分抽样的方法是可行的, 且有望在2030年前投入使用。**【翻译:胡怡思;审校:聂永刚】**

## INTRODUCTION

Several indicators monitor progress toward the goals of the Convention on Biological Diversity (CBD) Global Biodiversity Framework (GBF). The framework aims to “halt and reverse biodiversity loss to put nature on a path to recovery” by 2030 and to have biodiversity “valued, conserved and wisely used, maintaining ecosystem services, sustaining a healthy planet and delivering benefits essential to all people” by 2050 (https://www.cbd.int/gbf/vision). These aims are underpinned by 4 goals. Goal A includes 3 main ambitions for species: to halt human induced extinctions of known threatened species; to dramatically reduce extinction risk; and to ensure that “the abundance of native wild species is increased to healthy and resilient levels.” The International Union for Conservation of Nature (IUCN) Red List Index (RLI) has been adopted by the CBD as a headline indicator for tracking extinctions and extinct risk (Convention on Biological Diversity [CBD], 2022). However, at present, there is no indicator tracking species recovery despite this being enshrined in the CBD's mission. Indicators, such as the Living Planet Index (currently a component‐level indicator for Goal A), measure increase in abundance over time (Geldmann et al., 2023) and hence provide a measure of population recovery but are silent as to whether populations have increased to “healthy and resilient levels” (McGowan et al., 2024).

This deficit is particularly important to remedy because the vast majority of the biodiversity that matters to people, and which is important for the correct functioning of ecosystems, is not threatened with global extinction. Therefore, tracking progress toward the 2050 mission of a healthy planet and addressing the broader context of both the CBD and the UN Sustainable Development Goals require a more inclusive perspective about the status of species that contribute to maintaining and supporting social–ecological systems.

Beyond tracking species recovery, it is also necessary to understand whether and how conservation efforts are contributing to those trends. This allows actors implementing the GBF (governments and nongovernmental organizations) to evaluate their actions and ensure that they are effective. Previous approaches to measuring the impact of conservation action on species (e.g., Hoffmann et al., 2015; Young et al., 2014) have tended to draw on the IUCN Red List and hence are constrained by the coarseness of the list's categories (Hoffmann et al., 2010) and by low risk of extinction being treated as an endpoint, whereas species with a low risk of extinction can still be some way from recovery (Grace, Akçakaya, Bennett, et al., 2021).

Recognizing the need for species conservation to aim beyond avoiding extinctions toward promoting recovery and the need for a general framework for evaluating conservation impact, the IUCN adopted Green Status of Species (GSS) as a global standard for measuring species recovery and for assessment of the role played by conservation in the recovery of species (IUCN, 2021). An index based on the GSS has been adopted as an indicator for multiple elements of the GBF, including as a component indicator for GBF Target 4 and as a complementary indicator for GBF Goal B (CBD, 2024). However, a methodology underpinning the index itself has not been published or elaborated previously.

We formally outlined the scientific foundation of the Green Status Index of Species Recovery (GSI) and explored its utility as an indicator of progress toward species recovery at the international, national, and programmatic levels. We proposed and demonstrated quantitative methods for incorporating uncertainties and disaggregating the global index to countries. Finally, we considered the strengths and assumptions of the GSI, its relevance to the GBF, future research needs, and practical actions required to operationalize it as a functional global index.

## The Green Status of Species

The GSS is used to assess 3 essential dimensions of recovery: viability, functionality, and representation. A species is fully recovered (or not depleted) if, in all parts (spatial units) of its indigenous range, it is viable (i.e., not threatened with extinction) and ecologically functional. These factors contribute toward a green score ranging from 0% to 100%, which shows how far a species is from its fully recovered state. This definition of *recovery* is ambitious by design. It is not expected and it is not a goal that all species will eventually fulfill this definition of full recovery; for many species, large areas of range have been irrevocably modified. Instead, this definition serves to standardize the assessment approach between species, to identify areas of recovery opportunity in the context of what has been lost, and to avoid shifting baselines (Papworth et al., 2009).

A GSS assessment not only reports how far a species is from being fully recovered but also estimates how conservation actions have affected the current status of the species and how the species’ status might change if conservation actions were to be halted or increased in the future. This is reflected in a set of conservation impact metrics based on scores calculated for different scenarios in the past and in the future (Table 1).

**TABLE 1:** The International Union for Conservation of Nature Green Status of Species (GSS) metrics and interpretation of each metric and its minimum and maximum possible values.

Metric	Interpretation of metric	Interpretation of minimum value (0%)	Interpretation of maximum value (100%)
Species recovery score (SRS)	How close the species is to being fully recovered	The species is extinct or extinct in the wild.	The species is fully recovered or not depleted; it is viable and functional in all parts of its range.
Conservation legacy	Contribution of past conservation actions to the recovery of the species	Past conservation did not affect recovery status of the species.	The species is fully recovered or not depleted but would have been extinct without conservation.
Conservation dependence	Expected change in the recovery status of the species in the short‐term future if all conservation actions were to cease today	The species does not depend on existing conservation measures, and cessation of measures is not predicted to affect the future recovery status of the species.	The species is fully recovered or not depleted but would go extinct in the near future if conservation ceased.
Conservation gain	Expected improvement in recovery status of the species in the short term if ongoing and planned conservation actions are implemented effectively	Planned and ongoing conservation actions are not predicted to affect the future recovery status of the species.	The species is currently extinct in the wild and will become fully recovered in the near future because of ongoing and planned conservation.
Recovery potential	The maximum plausible improvement in status of the species with sustained conservation efforts and conservation innovation over the long term (100 years)	Conservation (even if innovative and sustained) will not affect recovery status of the species.	The species is currently extinct in the wild and will become fully recovered in the long‐term future because of conservation.

Methods of GSS were developed by an international team under the auspices of the IUCN Species Survival Commission. The global standard (IUCN, 2021) and associated guidelines (IUCN Green Status of Species Working Group, 2024) drew from publications in which authors developed and tested the underlying methods (Akçakaya et al., 2018, 2020; Grace et al., 2019, 2022; Grace, Akçakaya, Bennett, et al., 2021; Grace, Akçakaya, Bull, et al., 2021; Stephenson et al., 2019). The completed GSS assessment of a species is published alongside its IUCN Red List assessment in the IUCN Red List.

## THE GSI

The derivation of the GSI is similar to that of the RLI (Butchart et al., 2005, 2007, 2025). Just as RLI tracks genuine deteriorations and improvements in the extinction risk of species over time, GSI tracks genuine changes in species’ recovery.

### Derivation and interpretation of GSI

The index is calculated for a point in time by first assessing the green status of each species at that time according to the GSS global standard (IUCN, 2021). This assessment involves calculating each species’ current green score, known as the species recovery score (SRS). The GSI is then calculated as the average of SRS values over all the species being assessed; it measures how close the species are, on average, to being fully recovered and ranges from 0% (all species are extinct or extinct‐in‐the‐wild) to 100% (all species are fully recovered or not depleted). An increase in GSI through time indicates more species moving toward full recovery.

For each species, the SRS is calculated with minimum and maximum values representing uncertainties (Akçakaya et al., 2018; IUCN, 2021). Uncertainty in the GSI is expressed with a lower and an upper bound, which are calculated as averages of the minimum and maximum SRS values of all species, respectively.

### An example of calculating GSI

**FIGURE 1:**
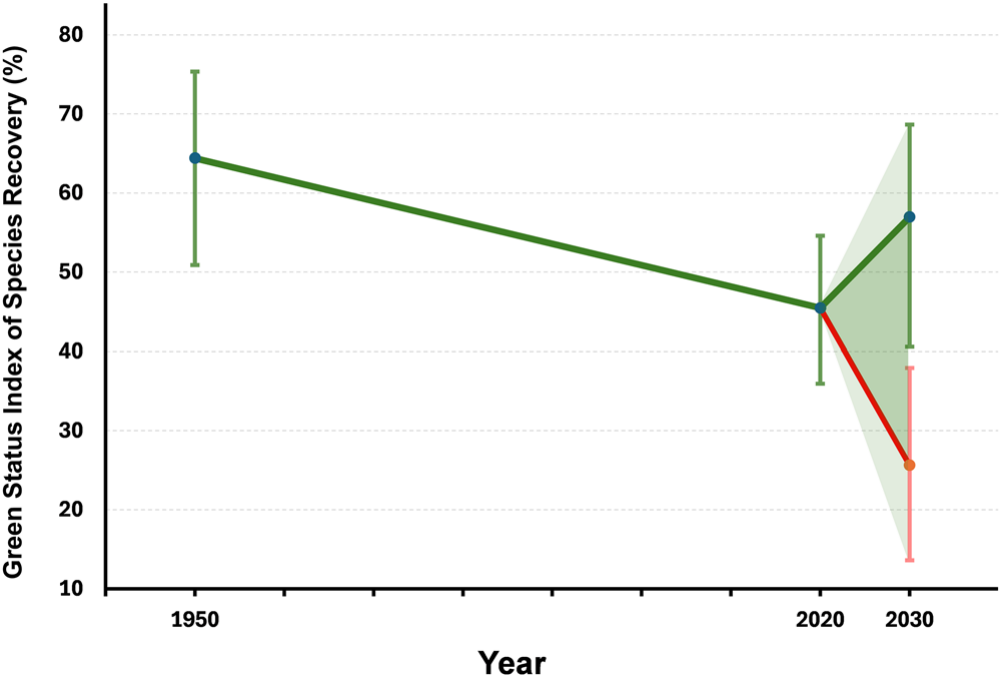
The Green Status Index of Species Recovery (GSI) over time for 165 species in Grace, Akçakaya, Bennett, et al. (2021). The 2020 value of the GSI is the average species recovery score, with lower and upper uncertainty bounds. The 1950 value is the retrospective GSI. The 2030 values are the leading indicators of the GSI, showing expected values in a future with conservation (green) and without conservation (red). The 165 species in this example were not sampled representatively; this figure is for illustrative purposes and does not represent global GSI.

We illustrate the derivation of the GSI based on the GSS assessments of 165 species included in Grace, Akçakaya, Bennett, et al. (2021). For this illustration, we selected all full species (not subspecies or subpopulations) assessed by Grace, Akçakaya, Bennett, et al. (2021) for which all green scores, including former state (described below), were calculated. The GSI in 2020 for these 165 species was 45%, with uncertainty bounds of 36–55% (Figure 1). Figure 1 is meant as an illustration of the GSI, not as the actual indicator because of the way species were selected (see “Selecting species” below).

### A leading indicator

A leading indicator is a metric that predicts the future trend in a variable of interest. Two of the 4 conservation metrics (Table 1), conservation dependence and conservation gain, are based on the difference between the SRS (i.e., current green score) and a green score calculated for the short‐term future (10 years) under 2 alternative scenarios: future with conservation (if ongoing and planned future actions are implemented effectively) and future without conservation (if all conservation actions were to cease, beginning today).

The data displayed in Figure 1 for 2030 give the average values of the green scores under these 2 scenarios for 165 species and the uncertainty bounds for each scenario. The difference between these 2 averages (57% for future with conservation and 26% for future without conservation) represents the potential contribution that conservation measures could make to the GSI in the next 10 years, depending on either the extent to which ongoing and existing conservation actions are maintained (and planned conservation actions are implemented) or the degree to which these actions cease or are withdrawn.

**FIGURE 2:**
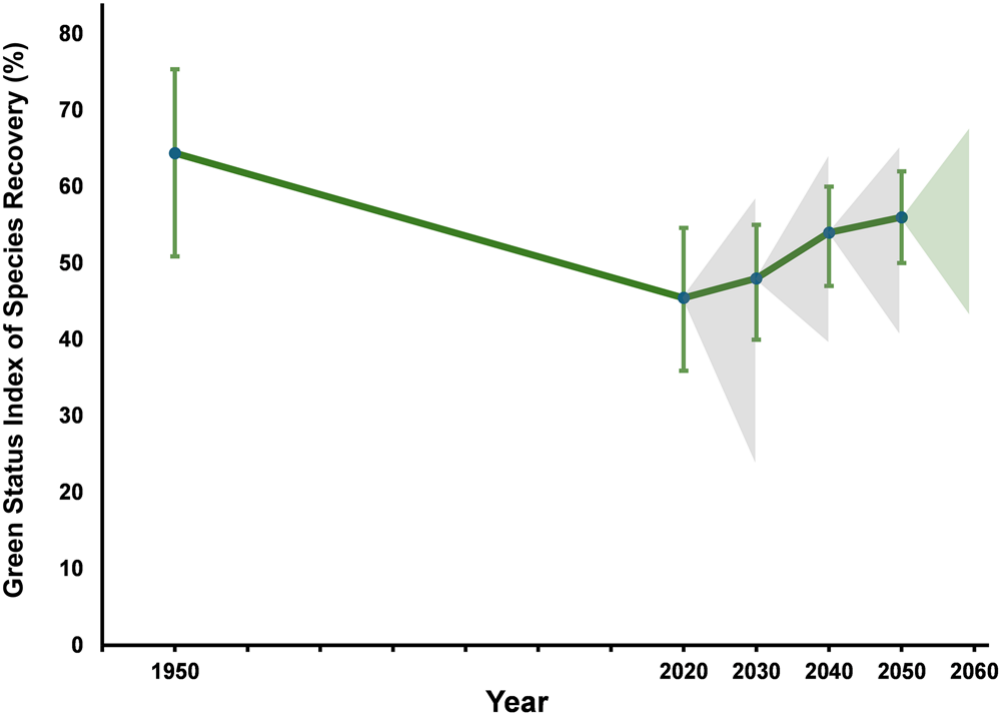
An illustration of what the Green Status Index of Species Recovery (GSI) might look like in 2050. For 1950, 2020, and 2020–2030 (gray triangle), data are for the 165 species in Grace, Akçakaya, Bennett, et al. (2021), as shown in Figure 1 (green, GSI over time; green triangle, leading indicator of GSI in 2050; gray triangles, past projections of GSI that are not relevant in 2050 except as a historical record of previous projections). Values after 2020 are hypothetical but account for retrospective assessments. The species included in this example were not sampled representatively; this figure is for illustrative purposes and does not represent global GSI.

Thus, the GSI not only measures how close a group of species currently is to being fully recovered but also functions as a leading indicator of species recovery: it is not only descriptive but also predictive. At every point in time, GSI will measure how close the species are to being fully recovered and project where the GSI is likely to be 10 years into the future with and without conservation (Figure 2). The predictive aspect of GSI (green triangle at the last GSI estimate in Figure 2) is important for conservation planning and for measuring the effectiveness of current and planned conservation. It will also draw attention to the importance of maintaining and implementing conservation measures in the near future. Over time, previous projections (gray triangles in Figure 2), although no longer relevant, function as a historical record of past conservation ambition, showing how effective planned conservation has been in the recent past.

### Retrospective assessments

Although an indicator is usually developed through repeated assessments over time, in some cases retrospective assessments are used to calculate a trend relative to the past, for example, the change in RLI for amphibians (Butchart et al., 2005) and corals (Carpenter et al., 2008). Because the GSS is rather new, the derivation of the GSI will require comparison with retrospectively determined values. The GSI has an advantage over the RLI in this regard because many GSS assessments also include a former state, for which the green score is calculated for 1950, a year selected as an approximate start of modern biodiversity conservation. Although for a minority of species the 1950 default is not used, if conservation for them did not start until later, it is assumed that their SRS would have been the same in 1950. In other words, GSI has a built‐in retrospective assessment. In Figures 1 and 2, the average green score in 1950 is shown (i.e., what the GSI for this set of species would have been at that time).

As repeat GSS assessments over time become the norm, the ability to track genuine changes in the GSI will be key. This will mean determining whether a change in a species’ SRS has been due to conservation interventions and changing pressures or whether they are not genuine changes, such as new information becoming available to the assessors since the previous assessment (which could, e.g., result in changes to the benchmark date or the delineation of spatial units); a change in taxonomy; an assessor error; and changes to the criteria or to the GSS guidance.

The process of distinguishing between changes that are genuine and changes that are not genuine is well established and elaborated for the RLI (Butchart et al., 2004, 2006, 2007; Hoffmann et al., 2011) and will be similar for the GSI. For example, a change in green score may be due to the state of a species changing from absent to present in a spatial unit between periods. On the one hand, this might be genuine, if a successful translocation has taken place. On the other hand, it might not be genuine if the species had always been present and inadvertently marked as absent. If a change in SRS is not genuine, its value in the earlier period will be retrospectively corrected.

### Conservation legacy

The change in GSI from 1950 (when it was retrospectively estimated to be 64% for our example set of species) to 2020 (45%) shows that this set of species moved further away from being fully recovered, despite conservation efforts. A reduction in recovery status is not surprising given the increasing threats to biodiversity over the last 70 years, but it does not mean that conservation measures since 1950 did not work. The impact of past conservation can only be calculated by developing counterfactual scenarios, which measure what the current state of these species would have been had there been no conservation in the past (Grace, Akçakaya, Bull, et al., 2021). Assessing this counterfactual is already a part of the GSS, via conservation legacy (Table 1). For the species in our sample, the counterfactual value of GSI in 2020 is 30%. In other words, without conservation, these species would have been, on average, about 15% further away from recovery (and thus 15% closer to extinction). Although the trend in conservation legacy is not proposed as a primary part of the GSI, it forms a valuable contextual addition for demonstrating the impact of conservation for a wide range of species for which quantitative impact evaluations are not available (Appendix S1).

## DISAGGREGATION OF THE GSI

Like the RLI, the GSI can be disaggregated to show trends for subsets of species (e.g., by ecosystem or threat). However, of greatest relevance to Parties to the CBD is the ability to disaggregate the GSI to produce national GSS indices. This can be achieved in 2 different ways. Although both approaches measure the contribution of conservation within the borders of a country to the recovery status of species that occur in that country, they measure different aspects of this contribution and are intended for different purposes. These 2 distinct disaggregation methods correspond to the 2 methods used to calculate national red‐list indices (Raimondo et al., 2023).

We defined *country‐level GSI* as a country's contribution to the global GSI. The sum of these values across all countries equals the global GSI value. The larger the number of countries a species occurs in, the smaller each country's contribution to the global indicator via that species will be. A small value of a country‐level GSI does not mean that the country is not contributing to the conservation of the species. It means that in a global context, the contribution is scaled according to the proportion of the species’ range that falls within its borders. This is analogous to the disaggregation of the RLI to countries (Rodrigues et al., 2014), called the “disaggregated global RLI” (Raimondo et al., 2023). It is intended for objective, international monitoring of the contribution of countries to global biodiversity conservation.

Conversely, we define the *national GSI* for a country as the GSI calculated at the national level, considering only the information (and the ranges of the species) within that country. This is analogous to the “national RLI” (Raimondo et al., 2023), which is based on national red‐list assessments (nationalredlist.org). Thus, it is intended for helping with national priorities and within‐country monitoring. The national GSI is not affected by the number of countries the species occurs in. A large national GSI means that the species are close to being fully recovered within that country, even if their ranges in that country are small in a global context. Like a national RLI, the national GSI would normally be based on national GSS assessments (but see Appendix S2 for an alternative). It would be simply the average of the SRS values calculated at the national level for all species in a national GSS assessment. The national GSI methods are described in Appendix S2. Because country‐level GSI is more immediately relevant to CBD monitoring, we focused on it below.

For the country‐level GSI, the disaggregation of global GSI starts with the basic building blocks of the GSI (i.e., the SRS for each species). First, the global SRS value of each species is disaggregated to countries:

(1) $$G_{C}=\frac{\sum_{s}\left(A_{\mathrm{SC}}W_{S}\right)}{W_{F}\times N}\times 100$$

where *G_C_* is the country‐level green score (SRS) for country *C*, *S* is each spatial unit, *W_S_* is the weight of the state in the spatial unit *S*, *W_F_* is the weight of the Functional state, *N* is the number of spatial units, and *A_SC_* is the proportion of the spatial unit *S* in country *C*. The weights are 0, 1, 2, and 3, for absent, present, viable, and functional, respectively, with default weights for states (IUCN Green Status of Species Working Group, 2024). The value of *G_C_* ranges from 0% (for a species extirpated in the country) to 100% (for a species endemic to the country that is fully recovered or not depleted).

Then, for each country, the disaggregated SRSs are averaged (over species) to calculate the country‐level GSI. In summary, the disaggregated value of the GSI for each country is simply the average, over all species in that country, of the disaggregated SRS values (each of which, in turn, is calculated using Equation 1).

Thus, the method of disaggregating SRS for one species requires only the global GSS assessment, which shows the state (absent, present, viable, or functional) in each spatial unit of the species, and a table showing the area of each spatial unit in each country. This table is used to calculate a matrix (**A_SC_**), which gives the proportion of the spatial unit *S* in country *C* (i.e., area of *S* within the borders of the country *C*, divided by the total area of *S*). Thus, for a given *S*, the sum of *A_SC_* for all countries equals 1.

The disaggregation calculations are based on the area of each spatial unit in different countries. This allows the calculations to be carried out with only a map of countries and the global GSS assessments. This is similar to the approach used to disaggregate the RLI (Rodrigues et al., 2014). In cases where a spatial unit spans multiple countries, this approach is based on an assumption of uniformity within a spatial unit. In other words, densities of the species and effectiveness of conservation measures are assumed to be similar across a spatial unit. For spatial units contained within one country, the result would be the same regardless of whether the calculations are based on the area or on some other variable.

### Examples of disaggregating the GSI to countries

We demonstrated the method for the disaggregation of the SRS of one species with 2 examples. The first is a simple, hypothetical example of a species that occurs in 6 spatial units across 3 countries (Appendix S2). We used the same case to illustrate both types of disaggregation, the country‐level GSI and the national GSI (Appendix S2; also available in a spreadsheet for explicitly showing the calculations; see link in Appendix S2). The second, a real case study based on the GSS assessment of saiga (Milner‐Gulland & Mallon, 2024), illustrates both types of disaggregation to countries (Appendix S3).

### Advanced properties of the GSI and its disaggregation

There are 4 additional, advanced aspects of the GSI. First, the state in each part of the range (spatial unit) can be assessed at a high resolution, with 10 discrete values instead of the 4 mentioned above (IUCN Green Status of Species Working Group, 2024), which allows GSI to track smaller changes in status in each spatial unit. Second, the number of values the GSS status can take is determined by the number of spatial units (whereas IUCN Red List status can only take 6 discrete values, regardless of the area of the species’ range). These 2 attributes allow the GSI to track incremental improvements. For wide‐ranging species that occur in many different ecological contexts, the range of green scores can be indistinguishable from a continuum, allowing GSI to reflect local and small improvements in status.

Third, the disaggregation formula for SRS (Equation 1) also applies to all the other green scores (counterfactual current, future with conservation, future without conservation, long‐term potential, and current baseline) and therefore can also be used to disaggregate the global values of the conservation impact metrics given in Table 1 (in addition to SRS). Although this is not the focus of the GSI as proposed here, it will be available to countries interested in these values. For example, considering the trend in conservation legacy mentioned above, a country could, in principle, determine its contribution to the counterfactual GSI value. Fourth, the global GSI can be aggregated based on national GSS assessments (which is possible for GSI but not for RLI). This is not a calculation recommended as an integral part of GSI, but it may be useful in special circumstances (Appendix S2).

## IMPLEMENTATION PLAN

The next steps for implementing the GSI are selecting species, making GSS assessments for each species, and processing of the assessment results, including uncertainties.

### Selecting species

Like the RLI, the GSI can be deployed 2 ways: using comprehensively assessed groups of species and using a sampled approach similar to that developed for the RLI (Baillie et al., 2008; Henriques et al., 2020). Realistically, the first option is years away, so the GSI is proposed initially as a sampled index.

How species are selected for inclusion in the sample depends on the priorities with respect to the use of the indicator. For the GSI to support monitoring for the GBF, the priority would be to ensure the ability of countries to monitor their contributions to global species recovery based on the disaggregation method presented above. In this case, the species would be selected randomly but stratified by regions and subject to constraints of each country being sufficiently represented in the sample. The constraints would be based on information from the IUCN Red List, including the list of the countries a species occurs in and the proportion of its range in each country. This sampling method is being developed and will be presented in a future publication following testing.

### Required information for GSS assessments included in GSI

Developing the GSI requires a GSS assessment of each sampled species. These will be standard GSS assessments, but they will include the following information, which is normally recommended but not required, for all GSS assessments: a table showing the area in square kilometers of each spatial unit in each country (example in Appendix S3); in addition to the SRS, assessments of former, future‐with‐conservation, and future‐without‐conservation green scores; if a repeat assessment, a determination of whether the change from the previous assessment is genuine (i.e., resulting from changes in the actual recovery status of the species due to changing threats, natural conditions, or conservation measures) or not genuine (reason for change is coded); and if the change is not genuine, a retrospective corrected assessment to adjust previous assessments according to the current knowledge, definitions, and settings.

### Incorporating uncertainties in the global index

As described above, the minimum and maximum GSI values are calculated as the average of the minimum and maximum values, respectively, of the SRS for each species. This results in error bars around the average of the best values for each species (Figures 1 & 2).

This approach is based on the assumption of complete correlation (dependence) among species with respect to uncertainty (i.e., that the bounds of aggregate uncertainty involve the SRS being either underestimated for all assessed species or overestimated for all assessed species; thus, under‐ and overestimated values cannot cancel out each other). This is a reasonable and precautionary assumption in that it does not underestimate uncertainty. However, uncertainty bounds with this approach may be large for groups that include a large number of species in the Indeterminate category (when the difference between the maximum and minimum values of the SRS is >40%; IUCN, 2021). One possible modification is to exclude the indeterminate species from the GSI, which would narrow the uncertainty range of the index and would be consistent with the RLI, which excludes data‐deficient species.

## DISCUSSION

The GSI fills an important niche in measuring biodiversity trends, by focusing on the overall progress toward recovery of a given set of species over time. The methods we described calculate the GSI as the arithmetic average of the results of GSS assessments of a group of species; display trends in GSI based on repeated assessments over time; incorporate uncertainties; and disaggregate the global index to countries. Our application of GSI to a small group of species that had been initially assessed was a test of the underlying global standard. However, a large‐scale test of the proposed GSI will require a systematically selected set of species, as specified in the implementation plan outlined above.

### Strengths of the GSI

The GSI exhibits many key attributes of an effective global indicator (Jones et al., 2011; Watermeyer et al., 2021). Its fundamental objective (measuring trends in biodiversity) and its means objective (monitoring how close a group of species is to being fully recovered) are explicit and closely aligned with global biodiversity goals, such as those of the GBF (discussed in “Alignment with the global biodiversity framework”). Like the RLI, it is applicable to all species except microorganisms; thus, it can provide information about diverse taxonomic groups. The scientific basis and quantitative methods underlying the GSI are well established in a global standard (IUCN, 2021) and in the peer‐reviewed literature (Akçakaya et al., 2018, 2020; Grace et al., 2019, 2022; Grace, Akçakaya, Bennett, et al., 2021; Grace, Akçakaya, Bull, et al., 2021; Stephenson et al., 2019); thus, it can reliably inform about the status and trends of biodiversity. Methods exist for propagating uncertainties in GSS assessments (Akçakaya et al., 2018; IUCN, 2021) and for incorporating them into the GSI (this paper). The GSS assessments, including the data they are based on, are openly available in the IUCN Red List. Crucially, as an IUCN metric incorporated in a globally respected IUCN knowledge product, the GSS is internationally recognized and has a strong institutional home to ensure that it is maintained, a home where it can draw on the tremendous expertise of the IUCN Species Survival Commission and the wider IUCN Red List partnership.

The GSS itself also has many attributes that position the resulting GSI as highly complementary to the RLI. The GSI can track changes in the Species Recovery Category over time (as the RLI does for extinction risk categories), but the numerical scores in the GSS also allow tracking incremental changes and avoid the need for weighting of categories (RLI requires category weights). In addition, by explicitly considering the whole indigenous range of a species, the GSS recognizes variation in species’ status across its range and hence guards against recovery of small, highly protected populations masking range‐wide declines. As such, it is suitable for tracking the status of both widespread and abundant species and narrowly endemic or threatened species.

Indicators of progress toward global goals should ideally not just passively track progress at a global level. To help drive the change needed to halt and reverse the loss of biodiversity, these indicators should be capable of use in a predictive and adaptive way (Nicholson et al., 2012). The GSI offers one major advantage over many other indicators in this regard, in that it has an in‐built predictive capability that forecasts future trends in scenarios both with and without planned and ongoing conservation action. Thus, the GSI can provide an objective measure of the effectiveness of conservation actions in terms of their contribution to species recovery. The GSI for a comprehensively assessed group of species (at country or taxonomic levels) would provide a robust picture of species’ recovery status and the past and potential impact of conservation management actions. In turn, the conservation impact metrics (Table 1) can be used as benchmarks against which achievement toward halting the decline of nature is assessed as species are reassessed in the future.

### Alignment with the global biodiversity framework

The GBF sets out 4 goals for 2050, with 23 targets to measure progress toward those goals in 2030. This progress is monitored with different types of indicators. Headline indicators are intended to represent a minimum set that captures the overall scope of the goals and targets of the GBF. Component indicators are optional indicators that together with the headline indicators cover all components of the goals and targets of the GBF. Complementary indicators are optional indicators for thematic or in‐depth analysis of each goal and target. The GSI is currently listed as a complementary indicator for Goal B (“prosper with nature”). Goal B includes a focus on maintaining and restoring ecosystem functions and services, which aligns with the GSS focus on the recovery of species to ecologically functional levels. Increasing values of the GSI indicate an overall increase in the viability and ecological functionality of species. The GSI therefore provides the first global metric for species recovery that considers not only the viability of the species but also its contributions to ecosystem function and its representation in all ecological settings of its indigenous range.

Although the GSI is not currently listed as an indicator for Goal A (“protect and restore”), it would function as a reliable means for measuring progress toward Goal A's aspiration of increasing the abundance of native wild species to healthy and resilient levels. McGowan et al. (2024) argue that *healthy and resilient levels* could imply that species’ populations are healthy throughout their extant native range and that reintroduction to, or natural recolonization of, as much of the former range as possible is essential to meet this goal. Taken together, this would be equivalent to a species being at least viable and, ideally, functional in each part of its indigenous range, as defined in the GSS.

The GSI could also usefully track progress toward GBF Target 4, where it is currently indicated as a component indicator. One of 3 components of target 4 is “management actions need to be taken to halt human‐induced extinctions by 2030 and to reduce extinction risk, in particular for threatened species.” The disaggregation methods we developed may be used to produce a version of the GSI showing trends of species requiring specific and targeted actions (or where trends in conservation legacy are aided by species‐targeted actions), such as those called for under this target. Other relevant targets, such as 5 and 9 (related to sustainable use of species), could also benefit from the GSI. Related to this, the European Union Pollinator Monitoring Scheme has recently proposed GSS as one of 2 indicators for measuring trends of pollinator species (Potts et al., 2024).

Like countries, many conservation organizations are also keen to quantify their contributions toward the GBF. In addition, GBF Target 15 calls for businesses to increase their positive impacts. The GSS can be calculated at the programmatic level to support the attribution of change in biodiversity to a particular program in the context of wider trends in species recovery (Young et al. in review). This calculation will allow GSI to also monitor conservation impacts of organizations and companies.

### Challenges with implementing the GSI

Since 2004, RLIs for species groups with comprehensive assessments repeated over time have been calculated for birds (8 time points), mammals (2) (Hoffmann et al., 2011), amphibians (3) (Luedtke et al., 2023), corals, conifers, and cycads. Sampled RLIs are also available for a handful of groups. Collectively, these RLIs represent ∼25,000 species that have been assessed on the Red List more than once. These assessments represent a considerable investment of time and resources from several institutions and thousands of individual contributors. The GSS is still relatively new, with 115 assessments published on the IUCN Red List website. Hence, GSI will require a considerable scaling up of effort. The most likely scenario is that GSI will rely on a sampled approach, until entire taxonomic groups can be assessed. Although we cannot yet state with confidence what sample size would be required to also ensure the GSI can be suitably disaggregated to the national level, experience with sampled RLI (e.g., Baillie et al., 2008; Henriques et al., 2020) suggests that the number is likely to be within the target of 7000 species assessed with the GSS, indicated in the IUCN Red List Strategic Plan to be achieved by 2030.

This discussion brings up the issue of the trade‐off between automated and labor‐intensive biodiversity assessments. On the one hand are calls for assessments based on technologies, such as remote sensing and AI, and on statistical approaches to uncover relationships between biodiversity and data that can be obtained by these technologies without spending time and effort on the ground. On the other hand, conservation planning demands on‐the‐ground, species‐specific, and location‐specific information and expertise. The GSS, and the other IUCN knowledge products in general, balances the use of both of these approaches and is a good compromise between desk‐based and on‐the‐ground analyses. They use remote sensing data and other databases where available and relevant and employ platforms (such as sRedList [Cazalis et al., 2024]) that facilitate and automate much of the data preparation and analysis. However, they also filter the information through local and taxonomic expertise of the thousands of assessors, reviewers, and contributors. This combination of technology‐assisted assessment and the mobilization of worldwide expertise strikes a balance between capacity, data availability, and simplicity without compromising the scientific robustness and conservation relevance.

The methods we presented here for GSI represent an indicator of species recovery aligned with the goals, targets, mission, and vision of the GBF. We expect that this indicator will be operational by 2030 and will contribute to reports on progress toward relevant targets.

## Supporting information



Supplementary Appendices
