# Supplementary material for: A global indicator of species recovery
Source: Conserv Biol. 2025 Jun 9;39(5):e70077. doi: 10.1111/cobi.70077 (PMC12451508; doi:10.1111/cobi.70077)
Supplement: Supplementary file 1 — Supplementary Appendices [file COBI-39-e70077-s001.pdf]

Supplemental Materials for

A Global Indicator of Species Recovery

H.R. Akçakaya, M. Hoffmann, E.J. Milner-Gulland, M.K. Grace, B. Long

Conservation Biology (2025)

Appendix S1: Actual and counterfactual GSI

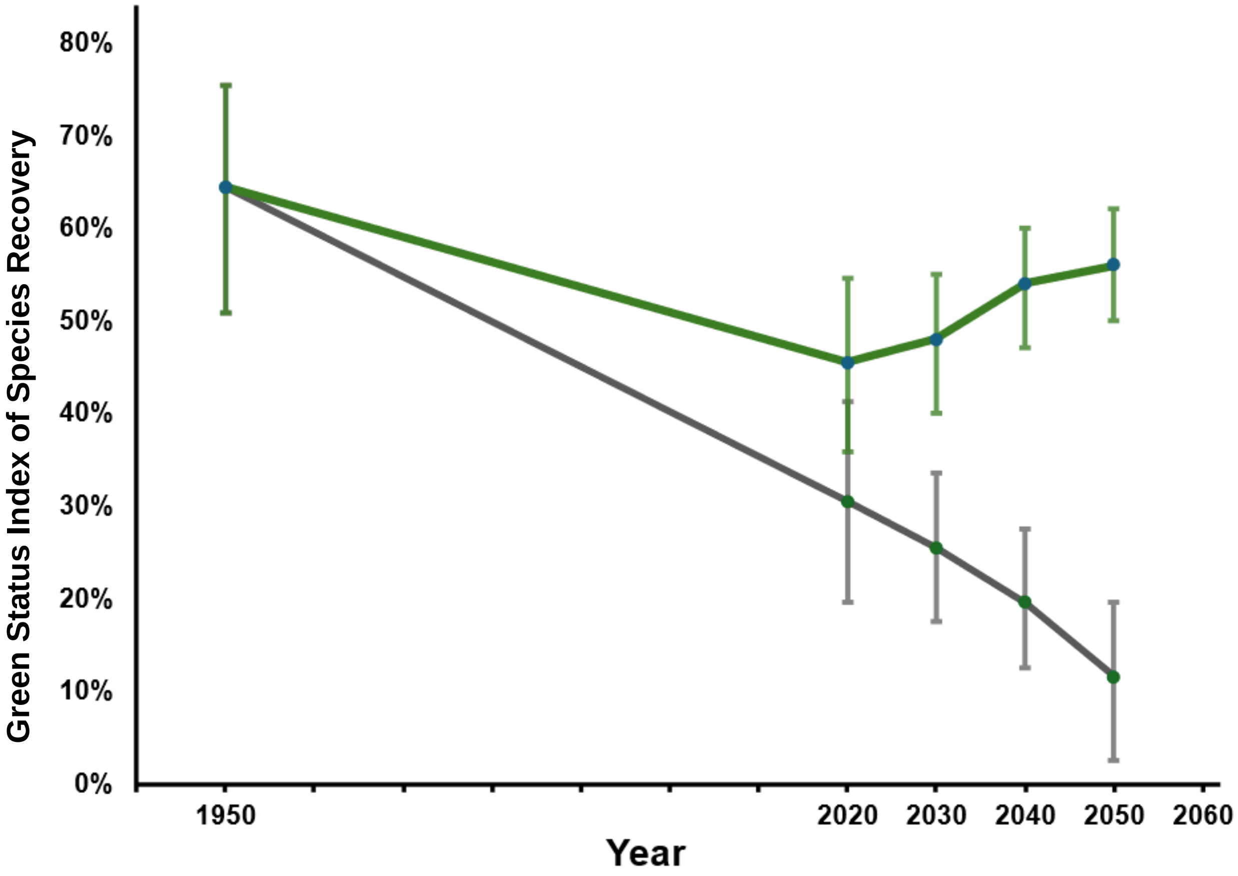

Appendix S1. Actual and counterfactual GSI. The green line shows the same information as in Figure 2. The gray line is the Counterfactual Current trajectory, showing what the GSI values would have been if no conservation had taken place since 1950. The values for 1950 and 2020 are the data for 165 species in Grace et al. (2021). The values after 2020 are hypothetical. The difference between the two curves shows the average Conservation Legacy through time for the assessed species.

Appendix S2: Calculation of the National Green Scores

As described in the main text, we define two types of national indicators based on the Green Status Index of Species Recovery (GSI). The ***Country-level GSI*** as a country's contribution to the global indicator, and is analogous to the disaggregation of the Red List Index to countries (Rodrigues et al. 2014; called "disaggregated global RLI" in Raimondo et al. 2023). The ***National GSI*** for a country is calculated at the national level, considering only the information (and the ranges of the species) within that country, and is analogous to the "national RLI" as defined by Raimondo et al. (2023), which is based on national red list assessments (nationalredlist.org). As discussed in the main text, these two indicators have different purposes (Table S1).

Both of these are calculated as the arithmetic average, over all species in the country, of the corresponding Species Recovery Scores (the current Green Scores): the Country-level Green Score (G_C) and the National Green Score (NG_C). As described in the main text, the former of these, the Country-level Green Score (G_C) is disaggregated from the global SRS using equation 1.

Table S1. Comparing the two methods of disaggregating the SRS (Species Recovery Score), which is the current Green Score of one species.

	**Country-level Green Score (G_C)**	**National Green Score (NG_C)**
Used for	Country-level GSI	National GSI
Measures…	…the contribution of a country to the global SRS value of the species	…the SRS at the national level, analogous to a national Red List assessment
Formula[1] for country *C*	$G_C = \frac{\sum_s (A_{SC} W_S)}{W_F \times N} \times 100$ (Equation 1)	$NG_C = \frac{\sum_s (A_{SC} W_S)}{W_F \times \sum_S A_{SC}} \times 100$ (Equation 2)
Minimum value	0% (for a species extirpated in the country)	0% (for a species extirpated in the country)
Maximum value	100% (for a fully recovered or non-depleted species that is endemic to the country).	100% (for a species whose population within the country is fully recovered or non-depleted).
Relation to the global SRS	The global SRS is the sum of the G_C values over all countries.	The global SRS is the weighted-average of the NG_C values, weighted by the country-level SU-equivalents, $\sum_S A_{SC}$.

[1] *S* is each spatial unit, W_S is the weight of the state in the spatial unit (SU), W_F is the weight of the Functional state, *N* is the number of spatial units, and A_{SC} is the proportion of the spatial unit *S* in country *C*. The weights are 0, 1, 2, and 3, for Absent, Present, Viable, and Functional, respectively, with default weights for states.

The National Green Score (NG_C) would normally come from a national assessment undertaken independently by a national group (just like the national Red List category of a species). However, it can also be disaggregated from the global value (unlike a national Red List category), if there are no taxonomic differences (i.e., the taxonomy used for global and national assessments is the same). A country may choose to use the disaggregated value (instead of doing a national assessment) because it is faster. Thus, to allow countries a choice, we present the disaggregation method below.

The two methods of calculating the SRS at the national level are compared in Table S1, and demonstrated with a hypothetical example in Table S2. Also, the Saiga case study (Appendix 2) demonstrates the calculation of both of these values.

Table S2. An example of calculating Country-level Green Score (*Gc*) and the National Green Score (*NGc*) for a hypothetical species that has 6 spatial units in 3 countries (see also the spreadsheet version, and the demonstration in Appendix 2 for a real species).

Spatial Unit (SU)	W_S	A_{SC}			$A_{SC} \times W_S$		
		Country A	Country B	Country C	Country A	Country B	Country C
SU-1	2 (Viable)	100%	0%	0%	2	0	0
SU-2	1 (Present)	70%	0%	30%	0.7	0	0.3
SU-3	0 (Absent)	0%	50%	50%	0	0	0
SU-4	2 (Viable)	60%	20%	20%	1.2	0.4	0.4
SU-5	3 (Functional)	30%	0%	70%	0.9	0	2.1
SU-6	3 (Functional)	0%	0%	100%	0	0	3
Total (SU-equivalents)=		2.60	0.70	2.70			
**Country-level Green Score** (*Gc*); contribution to the global Green Score					26.7%	2.2%	32.2%
**National Green Score** (*NGc*); Green Score calculated at the national level					61.5%	19.0%	71.6%
GNc * SU-equivalents					1.6000	0.1333	1.9333
Global Green Score (*G*)					61.1%		
Sum of *Gc* over all countries (sum of the contributions of all countries)					61.1%		
Weighted average of *NGc* over all countries					61.1%		

In the hypothetical example of Table S2, Country C includes more Spatial Units than other countries (2.7 SU-equivalents out of 6), and more of the SUs that are Functional (SU-5 and SU-6). Therefore, it has the highest values for G_C and NG_C. The largest contributions to its G_C come from the two Functional SUs. The maximum possible improvements are given in the spreadsheet version of this table.

National GSS assessments vs. GSI disaggregation

The National Green Scores for a given species and country that is generated in a national GSS assessment may differ from the values for that same species and country calculated using Equation 2 in Table S1. There may be various reasons for such a difference. One reason may be that, if a spatial unit extends to two or more countries, the distribution of the species and the effectiveness of conservation actions are not uniform across the spatial unit. For example, conservation within a spatial unit is much more effective in one country than in the other countries that the spatial unit covers. Note that this issue is minimized if most SUs do not overlap country borders. Hence, the global standard (IUCN 2021) states:

> To the extent possible, the species' range considered in a regional assessment should involve one or more of the spatial units of the global assessment in their entirety. […] Including whole spatial units (of the global assessment) in regional assessments will make it possible to combine results of two or more regional assessments, and therefore facilitate the information flow from regional to global assessments.

Obviously, this is easier for a regional assessment based on, for example, a biogeographic region, than one based on countries.

Another reason may be a difference in definition and delineation of spatial units between the national and the global assessment. The global standard (IUCN 2021) strongly recommends that "regional assessments (including national assessments) are done only after the first two steps of the global assessments are completed: determining the indigenous and expected additional range, and delineating spatial units." This would greatly reduce the differences between the metrics calculated at the global level and those aggregated from the national assessments. Despite this, global and national assessments may differ, especially for migratory species that have spatial units based on migratory pathways (e.g., flyways) in the global assessment, and that have only breeding (or only non-breeding) populations in some countries.

A third reason may be differences in scoring the counterfactual and future scenarios in a given spatial unit that overlaps multiple countries. To minimize such differences, it's important to remember that these scenarios in a national GSS assessment are supposed to include the effects of all conservation actions within the borders of the country, not just those that are part of national conservation programs.

The potential effect of such differences at the global scale can be examined by aggregating the National Green Score values coming from national assessments. In other words, NG_C values obtained in national assessments can be combined to calculate an aggregated global Green Score for each species. The difference between the actual global value and the one aggregated from national assessments allows determining the sensitivity of the global values to different definitions and assumptions used at the national and global levels.

The aggregation is done in two steps. First, given the National Green Score for a country (NG_C), the country-level Green Score is calculated as

$$G_C = NG_C \cdot \sum_S A_{SC} / N \qquad \text{Equation 3}$$

Second, the calculated G_C values are summed over countries to calculate the aggregated global Green Score. Such aggregation of national assessments is not possible for the Red List Index, but the GSS formula, based only on simple arithmetic operations, allows this calculation.

Marginally-occurring species in country-level index values

The country-level GSI values may be artificially low when averaged over all species that occur within the country, if the set of species includes many that occur only marginally in the country (these would be categorized as Not Applicable in a national Red List assessment). Whether these species should be excluded from the average remains to be decided. If they are to be excluded, this can be done by using the country weights, $\sum_S A_{SC}$. If, for a given species, this sum for a country *C* is less than, for instance, 1%, then that species can be excluded from the calculation of the Country-level GSI values.

Excluding marginal species means that the Country-level GSI values no longer add up to the global value. In order to keep the global value to be a sum of Country-level GSI values, the A_{SC} values for a given species can be modified for all countries as follows. If the sum of A_{SC} as for a country *C* is less than, for instance, 1%, then that country is removed from the calculation, and the A_{SC} values are recalculated for the remaining countries so that the sum of A_{SC} for any spatial unit (*S*) across all remaining countries is equal to 1.0.

Appendix S3. GSI case study: Saiga

The disaggregation of the GSS metrics to countries is demonstrated below, with the methods applied to the GSS assessment of Saiga (*Saiga tatarica*).

Step 1. Calculating A_{SC} (proportion of the spatial unit S in country C):

Using the Saiga range shape file downloaded from iucnredlist.org, and a shape file of country borders, the areas (in km^2) of each spatial unit (SU) in each country were calculated (Table S3).

This process required several manual corrections, which were made based on the descriptions of the SUs in the GSS assessment. First, one SU ("Mongolia") consisted of two separate polygons, which needed to be identified and merged. Second, the resulting polygons had to be matched to the SUs defined in the text because the shapefile polygons did not have an attribute indicating which SU they belonged to. Third, two of the SUs that have been assessed as Absent did not exist as polygons because they were extirpated. These SU were added to the table, and a nominal value was added for each to represent its area (Table S3).

Table S3. The areas (km^2) of each SU of *Saiga tatarica* in each country within its range

**Spatial unit**	**Country**					
	China	Kazakhstan	Mongolia	Russia	Ukraine	Uzbekistan
Betpak-dala	0	432219	0	0	0	0
China	0.001*	0	0	0	0	0
Mongolia	0	0	8538	0	0	0
Russia	0	0	0	34711	0	0
Ukraine	0	0	0	0	0.001*	0
Ural	0	95331	0	14724	0	0
Ustiurt	0	120270	0	0	0	43807

*A nominal value is added for SUs that are Absent.

In this case, each SU assessed as Absent was fully contained within one country, so the area entered for them would not change the result regardless of the numerical value used. If an Absent SU spanned multiple countries, a different (relative) value would be needed for each country. One option is that these are the areas of the species' range in each country before extirpation. This option may be more relevant for calculating SRS. However, for assessing conservation impact, areas of expected reintroduction or natural expansion may be more relevant.

Based on the calculated areas, the proportion of each spatial unit *S* in country *C* (A_{SC}) is calculated as the area of the spatial unit *S* within the borders of country *C*, divided by the total area of the spatial unit *S* (Table S4).

Table S4. Proportion of each spatial unit *S* of *Saiga tatarica* in each country *C* (A_{SC}), based on the areas in Table S3.

Spatial unit (S)	Country (C)						Total
	China	Kazakhstan	Mongolia	Russia	Ukraine	Uzbekistan	
Betpak-dala	0	1.000	0	0	0	0	1.0
China	1.000	0	0	0	0	0	1.0
Mongolia	0	0	1.000	0	0	0	1.0
Russia	0	0	0	1.000	0	0	1.0
Ukraine	0	0	0	0	1.000	0	1.0
Ural	0	0.866	0	0.134	0	0	1.0
Ustiurt	0	0.733	0	0	0	0.267	1.0
$\sum_S A_{SC}$	1.000	2.599	1.000	1.134	1.000	0.267	

Step 2. Calculating Green Scores for each country

The Current state of each SU is downloaded from the Spatial Units section of the Saiga GSS assessment, and converted into the default weights (0 for Absent to 3 for Functional; Table S5). Combining the data in Table S4 (A_{SC}) and Table S5 (W_S), green scores are calculated (Table S6) for min, best, and max values of W_S, for each country, and for both types of disaggregation to countries (G_C and NG_C). The formulae for these calculations are given in Table S1 (Appendix 1). The sum of Country-level Green Scores (G_C) across countries, and the weighted average of National Green Scores (NG_C) are calculated and confirmed that they are equal to the global value of Species Recovery Score (SRS). Note that the Current green score is defined as the SRS.

Table S5. The weights representing the State of each spatial unit for the Current Green Score. The weights are 0 (Extinct), 1 (Present), 2 (Viable), and 3 (Functional).

Spatial unit	W_S for Current		
	Min	Best	Max
Betpak-dala	2	3	3
China	0	0	0
Mongolia	1	1	2
Russia	1	1	2
Ukraine	0	0	0
Ural	2	3	3
Ustiurt	1	2	3

Table S6. The disaggregation of the Species Recovery Score of *Saiga tatarica* to countries. The data in Tables S4 and S5 are combined using the two equations in Table S1 for the Country-level Green Score (G_C) and the National Green Scores (NG_C).

Type of disaggregation	uncertainty range	**China**	**Kazakhstan**	**Mongolia**	**Russia**	**Ukraine**	**Uzbekistan**	Sum	Weighted average
**Country-level Green Score** (G_C)	min	0.0%	21.3%	4.8%	6.0%	0.0%	1.3%	33.3%	
	best	0.0%	**33.6%**	**4.8%**	**6.7%**	0.0%	2.5%	47.6%	
	max	0.0%	37.1%	9.5%	11.4%	0.0%	1.3%	61.9%	
**National Green Score** (NG_C)	min	0.0%	57.3%	33.3%	37.3%	0.0%	33.3%		33.3%
	best	0.0%	**90.6%**	**33.3%**	**41.2%**	0.0%	**66.7%**		47.6%
	max	0.0%	100%	66.7%	70.6%	0.0%	100%		61.9%

Step 3. Interpreting the disaggregated Species Recovery Scores (Table S6)

At the global level, the species is Largely Depleted (LD); the largest contribution to its Species Recovery Score (48%) is from Kazakhstan (34%) with smaller contributions from Russia and Mongolia. At the national level, the species is Slightly Depleted (SD) in Kazakhstan, Moderately Depleted (MD) in Uzbekistan, Largely Depleted (LD) in Russia and Mongolia, and Regionally Extinct (RE) or extirpated in China and Ukraine.

Step 4. Calculating the GSI for a country

The country-level GSI is calculated as the arithmetic average of the species-specific SRS values for all species in that country.
